# The application of peripheral blood immune profiling in personalized treatment of locally advanced and advanced lung cancer: a nomogram approach

**DOI:** 10.3389/fonc.2025.1642829

**Published:** 2025-09-01

**Authors:** Tianwen Yin, Yikun Li, Qixin Sun, Qipeng Yuan, Shan Zhu, Jinming Yu, Tao Zhang, Feifei Teng, Chuanwang Miao

**Affiliations:** ^1^ Cancer Center, Union Hospital, Tongji Medical College, Huazhong University of Science and Technology, Wuhan, Hubei, China; ^2^ Institute of Radiation Oncology, Union Hospital, Tongji Medical College, Huazhong University of Science and Technology, Wuhan, China; ^3^ Hubei Key Laboratory of Precision Radiation Oncology, Wuhan, China; ^4^ Department of Radiation Oncology, Shandong Cancer Hospital and Institute, Shandong First Medical University and Shandong Academy of Medical Sciences, Jinan, China; ^5^ School of Clinical Medicine, Shandong Second Medical University, Weifang, China; ^6^ Department of Graduate, Shandong First Medical University and Shandong Academy of Medical Sciences, Jinan, China; ^7^ Department of Radiation Oncology, Shandong Provincial Otorhinolaryngology (ENT) Hospital, Shandong University, Jinan, Shandong, China

**Keywords:** lung cancer, immunotherapy, lymphocyte subsets, nomogram, biomarker

## Abstract

**Purpose:**

Immunotherapy has revolutionized the treatment of lung cancer, yet many patients experience limited or transient benefits. Identifying those most likely to benefit remains a critical challenge. This study aims to establish a predictive model based on peripheral blood lymphocyte subsets to evaluate treatment responses in locally advanced and advanced lung cancer patients receiving chemotherapy with or without immunotherapy.

**Methods:**

We prospectively enrolled 171 patients, peripheral blood lymphocyte subsets were analyzed pre-treatment, post-treatment, and at disease progression using flow cytometry, focusing on CD3^–^CD16^+^CD56^+^ cells, CD3^–^CD19^+^ cells, CD3^+^CD4^+^ T cells, CD4^+^/CD8^+^ T-cell ratio, and CD3^+^CD8^+^ T cells. We assessed correlations between these subsets and treatment efficacy and constructed a nomogram to predict outcomes.

**Results:**

Baseline lymphocyte profiles were closely associated with treatment responses. Elevated CD3^–^CD16^+^CD56^+^ cells, increased CD4^+^/CD8^+^ T cell ratio, and higher CD3^–^CD19^+^ cells correlated with favorable treatment outcomes, particularly in patients receiving combined therapy. Conversely, higher CD3^+^ and CD3^+^CD8^+^ T cell counts were linked to poorer short-term efficacy. A nomogram integrating five immune parameters achieved an area under the receiver operating characteristic curve (AUC) of 0.778, outperforming individual marker. In the combination therapy subgroup, a four-parameter model achieved an AUC of 0.725. Furthermore, baseline and progression-stage lymphocyte profiles in responder and non-responder cohorts, exhibit no significant differences, indicating stable immune parameters over the disease course.

**Conclusion:**

Peripheral blood lymphocyte subsets are promising non-invasive biomarkers for predicting treatment responses in locally advanced and advanced lung cancer patients, particularly with immunotherapy. The developed nomogram models enhance predictive accuracy, supporting personalized treatment decisions.

## Introduction

Lung cancer ranks among the most frequently diagnosed malignancies worldwide and continues to be a leading cause of cancer-associated mortality, accounting for 18.7% of all cancer-related deaths globally (1–3). While surgical resection is considered the cornerstone of curative treatment in early-stage disease, the aggressive nature of lung cancer often results in delayed diagnosis. More than 75% of patients are diagnosed at locally advanced or metastatic stages, rendering them ineligible for radical surgery and contributing to the overall poor prognosis. Despite the implementation of multimodal treatment approaches—including chemotherapy, radiotherapy, targeted therapy, and more recently immunotherapy—the five-year overall survival (OS) rate for lung cancer remains below 20%, with only marginal improvement observed over recent decades (4, 5). The recent introduction of immune checkpoint inhibitors (ICIs), such as agents targeting programmed cell death-1 (PD-1) or programmed death-ligand 1 (PD-L1), has significantly altered the therapeutic landscape (6, 7). By restoring T cell functionality and dismantling immune tolerance, these agents can initiate durable antitumor responses. Nonetheless, merely about 30% of patients achieve substantial and enduring benefits from ICIs, leaving most individuals confronted with adverse events, substantial financial burdens, and suboptimal clinical outcomes (8). Refining approaches to extend survival and pinpointing patient subgroups most likely to benefit remain central challenges in lung cancer treatment. Under these circumstances, determining which individuals stand to gain the most from immunotherapy is a critical priority for optimizing clinical outcomes. To this end, various biomarkers—such as tumor mutational burden (TMB) and PD-L1 expression—have been leveraged to guide efficacy predictions (9, 10). However, due to their invasiveness, spatial heterogeneity and limited reproducibility, it`s challenging to utilize them as dynamic, real-time predictors of efficacy in clinical practice.

Peripheral blood, reflecting the overall status of circulatory system, serves as a valuable resource for monitoring and forecasting disease trajectories during cancer treatment (11). Lymphocytes, as the primary cell type in peripheral blood, are integral to both innate and adaptive immunity, collaboratively contributing to antitumor activities (12). Classical lymphocyte subpopulations include T cells (CD4^+^ and CD8^+^), B cells, and natural killer (NK) cells, all of which contribute to controlling tumor growth (13). However, directly assessing the immune environment within tumor tissue, particularly through measuring tumor-infiltrating lymphocytes, poses considerable practical difficulties for many patients with advanced disease, given the inherent challenges of acquiring repeated tumor specimens (14).

The systematic anti-tumor therapies remain a cornerstone in managing locally advanced or advanced lung cancer. However, their biological effects are multifaceted (15). On the one hand, the cascade of molecular events triggered by chemotherapy-induced tumor cell apoptosis can enhance lymphocyte activation and proliferation, contributing to tumor eradication. On the other hand, the intrinsic vulnerability of rapidly renewing lymphocytes to cytotoxic agents leads to significant myelosuppression. This reduction in lymphocyte quantity and functional capacity manifests clinically as lymphopenia, ultimately undermining sustained tumor control (16, 17). Under circumstances where functional lymphocyte populations are insufficient, the addition of PD-1/PD-L1 inhibitors may fail to yield therapeutic advantages.

Recent studies have suggested that peripheral blood lymphocyte subsets correlate with the immune status of patients with cancer, and the analysis of lymphocyte subset can serve as a non-invasive tool for treatment efficacy and prognosis assessment in cancer patients. However, few studies have investigated the heterogeneity in the predictive value of different lymphocyte subsets for lung cancer treatment, especially across various subgroups, considering the impact of different treatment regimens and dynamic changes during treatment. A nomogram is a reliable and convenient tool for predicting patient outcomes in oncology. By quantifying and integrating multiple prognosis factors, it provides more accurate, individualized predictions compared to traditional single-marker approaches.

In this study, we collected baseline and follow up peripheral blood lymphocyte subset data from patients with locally advanced or advanced lung cancer. We analyzed their relationship with short term treatment response and prognosis in different patients’ subgroups, sought to determine whether lymphocyte subset counts could serve as reliable indicators for anticipating therapeutic benefit in patients undergoing systemic therapies, particularly those receiving chemo-immunotherapy. Additionally, we aimed to construct nomograms that could accurately predict clinical efficacy, providing valuable evidence for the use of lymphocyte subsets as biomarkers to predict the efficacy of anti-tumor therapies.

## Methods

### Patients

From May 2018 to February 2024, we prospectively enrolled 171 individuals at Shandong Cancer Hospital and Institute who had been diagnosed with lung cancer and received chemotherapy with or without the addition of PD-1/PD-L1 inhibitors (**Supplementary Figure S1**). Peripheral blood lymphocyte of enrolled patient’s pre-treatment, post-treatment and at disease progression were collected. Flow cytometry was used to assess key lymphocyte populations, such as CD3^–^CD16^+^CD56^+^ cells, CD3^-^CD19^+^ cells, CD3^+^CD4^+^ T cells, CD4^+^/CD8^+^ T cell ratio, and CD3^+^CD8^+^ T cells. Patient survival outcomes were monitored through a combination of scheduled clinic visits, monthly telephone assessments, and verification of mortality records. Primary treatment response was evaluated by two radiologists who were blinded to clinical data, and discordant cases were adjudicated by third expert with access-restricted PACS. Progression-free survival (PFS) was defined as the interval from the initiation of systemic anti-cancer therapy until either radiologically confirmed disease progression or death from any cause. After two treatment cycles, comprehensive radiological evaluations were performed using computed tomography (CT). Treatment responses were then categorized according to the Response Evaluation Criteria in Solid Tumors (RECIST) version 1.1. This scheme classifies outcomes into complete response (CR), partial response (PR), stable disease (SD), or progressive disease (PD). In this study, the response group included patients who achieved CR, PR, while the non-response group consisted of individuals exhibiting SD, or PD.

All patient-related information was fully anonymized to maintain privacy, and strict confidentiality protocols were adhered to throughout the research process. Before enrollment, each participant was thoroughly briefed on the objectives and methods of the study, and written informed consent was obtained from all participants.

### Criteria of inclusion and exclusion

The inclusion criteria included: 1. Age between 18 and 80 years; 2. Histopathological confirmation of lung cancer; 3. Exhibit an ECOG performance status score of 0–2, and possess an anticipated survival exceeding six months; 4. Have completed a minimum of two chemotherapy cycles; 5. Peripheral blood lymphocyte subset data available for baseline and at least one follow-up time point.

Exclusion criteria were:1. Presenting with any secondary primary malignancies; 2. Death, loss to follow-up, or discontinuation of standard treatments or scheduled evaluations; 3. Baseline lymphocyte data showed ≥20% missing values. 4. Use of any immunomodulators agents at any point during the treatment interval, including but not limited to thymopeptides or placental polypeptides.

### Flow cytometry detection method

A qualified flow cytometry specialist, who was blinded to the clinical data, performed the assays using a BD FACS Canto™ (10-color) flow cytometer (BD, USA). The BD Multitest™ IMK kit was employed with following fluorochrome conjugated monoclonal antibodies: CD3-FITC/CD8-PE/CD45-PerCP/CD4-APC and CD3-FITC/CD16^+^CD56-PE/CD45-PerCP/CD19-APC, along with the matched isotype controls and corresponding lysis solution. All steps followed the BD operating manual instructions.

### Sample collection and data acquisition

Fresh peripheral blood (2mL) was collected from each enrolled patient into K2EDTA Vacutainer tubes (BD, Cat# 367841) and processed within 4 hours. The well-mixed blood sample was combined with an appropriate volume of lysis solution and incubated at room temperature in darkness for 15 minutes. Prior to antibody staining, the samples were centrifuged (1500 rpm, 5 minutes), and the supernatant was discarded. Aliquots of 100 μL whole blood were incubated with 20 μL antibody cocktails for 15 min at 20 °C in darkness. Centrifugation at 300g for 5 min followed by two washes with phosphate-buffered saline (PBS). The final cell pellet was resuspended in 0.5 mL of PBS.

### Flow cytometry analysis

The samples were then analyzed on the BD FACS Canto flow cytometer. Lymphocytes were identified by side scatter (SSC-A) and forward scatter (FSC-A) parameters; CD45 positivity and scatter properties were then used for more precise gating, with at least 10,000 lymphocyte events acquired per sample. Each lymphocyte subset was further delineated based on fluorescence signals, and the percentage of each subset was recorded. Total T Cells (T): CD3^+^, Helper T Cells (Th): CD3^+^CD4^+^, Cytotoxic/Suppressor T Cells (Tc/Ts): CD3^+^CD8^+^, Natural Killer Cells (NK): CD3^−^CD16^+^CD56^+^, B Cells (B): CD3^−^CD19^+^. Samples with <90% CD45^+^ cell viability were excluded.

### Statistical analyses and nomogram construction

Baseline characteristics between response and non-response groups were compared using Chi-square tests or Fisher’s exact tests, as appropriate. Treatment efficacy in the overall cohort and subgroups was evaluated using t-tests. Logistic regression was employed to analyze the combined predictive indicators of lymphocyte subsets. The predictive capability of each subset was analyzed using receiver operating characteristic (ROC) curves, and optimal cut-off values were determined by maximizing the Youden index (sensitivity + specificity – 1). Patients were then stratified into high and low groups based on these thresholds.

Independent predictors of treatment response (P < 0.05 in multivariate logistic regression) were incorporated into a nomogram to estimate the probability of therapeutic efficacy. Model performance were assessed using the area under the ROC curves (AUC-ROC) and the concordance index (C-index) to discrimination. Calibration curves were generated to assess the agreement between predicted and observed treatment response, with mean absolute error (MAE) and calibration slope calculated to assess model fit. Internal validation was conducted using bootstrap resampling (1,000 iterations) to ensure model stability. Sensitivity and specificity trade-offs were examined, and optimal cutoff values for lymphocyte subsets were determined by maximizing the Youden index, a pre-specified approach in the study design.

To ensure model reproducibility, the predictive formulas derived from logistic regression were as follows:

Entire cohort: p = 1/(1 + exp(-(-12.4210771597087 + 0.111 * a + 0.065 * b + 0.164 * c + -0.019 * e + -0.507 * f)))
Immunotherapy combination subgroup (IO): p = 1/(1 + exp(-(-6.08574866155402 + 0.023 * a + 0.094 * c + 0.027 * e + -0.487 * f)))
Chemotherapy subgroup (Chemo): p = 1/(1 + exp(-(1.82821229336954 + 0.064 * b + -0.034 * c)))

Decision curve analysis (DCA) was performed to assess the clinical utility of the nomogram by quantifying net benefit across different threshold probabilities. All the statistical analyses were performed using R software (version 4.2.2) and SPSS 22. 0. A two-tailed P < 0.05 was considered statistically significant.

## Results

### Patient demographic and clinical characteristics

A total of 171 patients were included, comprising 133 males and 38 females. The baseline characteristics of patients are shown in **Table 1**. Of the enrolled individuals, 75 (43.86%) were histologically diagnosed with adenocarcinoma (ADC), representing the predominant histological subtype. ADC prevalence differed significantly between those who responded to treatment and those who did not (*p* = 0.004). According to the 8th edition of the American Joint Committee on Cancer (AJCC) staging criteria, 62 patients (36.26%) presented with stage III disease, and 109 (63.74%) had stage IV disease, showing a statistically significant distributional difference between the two groups (*p* < 0.001). Among the 39 patients (22.81%) who underwent immunohistochemical evaluation for PD-L1 expression, 20 (51.28%) tested positive while 19 (48.72%) were negative. No other baseline characteristic demonstrated a statistically significant relationship with treatment response (*p* > 0.05). In terms of therapeutic approaches, 90 patients (52.63%) received a combination of chemotherapy plus immunotherapy, and 81 (47.37%) received chemotherapy alone. The response rates did not differ significantly between these two treatment strategies (*p* = 0.117).

**Table 1:** Baseline characteristics of enrolled patients.

Variables	Total (n = 171)	Non-Response (n = 91)	Response (n = 80)	Statistic	*P* value
Sex, n(%)				χ²=1.05	0.306
Female	38 (22.22%)	23 (25.27%)	15 (18.75%)		
Male	133 (77.78%)	68 (74.73%)	65 (81.25%)		
Age, n(%)				χ²=0.05	0.828
≤ 65	114 (66.67%)	60 (65.93%)	54 (67.50%)		
>65	57 (33.33%)	31 (34.07%)	26 (32.50%)		
Histology, n(%)				–	**0.004**
SCC	38 (22.22%%)	12 (13.19%)	26 (32.50%)		
ADC	75 (43.86%)	42 (46.15%)	33 (41.25%)		
SCLC	52 (30.41%)	35 (38.46%)	17 (21.25%)		
Others	6 (3.51%)	2 (2.20%)	4 (5.00%)		
PDL1, n(%)				–	0.751
Negative	19 (48.72%)	10 (45.45%)	9 (52.94%)		
Positive	20 (51.28%)	12 (54.55%)	8 (47.06%)		
Stage, n(%)				χ²=22.85	**<0.001**
III	62 (36.26%)	18 (19.78%)	44 (55.00%)		
IV	109 (63.74%)	73 (80.22%)	36 (45.00%)		
ICI, n(%)				χ²=2.46	0.117
No	81 (47.37%)	38 (41.76%)	43 (53.75%)		
Yes	90 (52.63%)	53 (58.24%)	37 (46.25%)		
ECOG, n(%)				χ²=1.88	0.171
0-1	157 (91.81%)	86 (94.51%)	71 (88.75%)		
2	14 (8.19%)	5 (5.49%)	9 (11.25%)		

SCC, squamous cell carcinoma; ADC, adenocarcinoma; SCLC, small cell lung cancer. The numbers and percentages of each characteristic in the response and non-response groups were shown. Differences in each characteristic between the two groups were analyzed using χ²: Chi-square test, -: Fisher exact. *P* < 0.05 was considered statistically different.

Bold values indicate statistical significance (P < 0.05).

### Predictive value of baseline lymphocyte subsets for treatment efficacy

**Figure 1:**
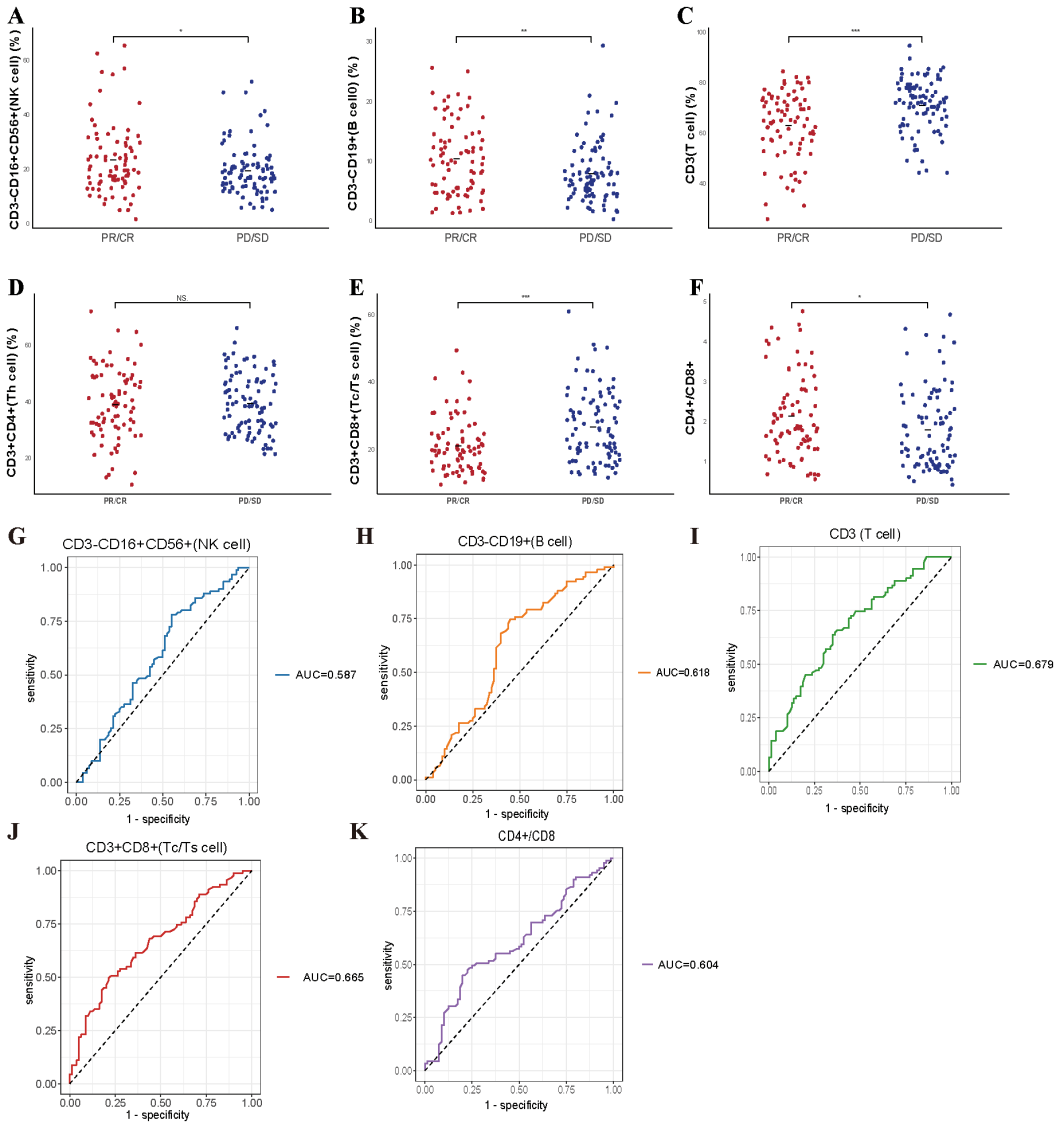
Predictive value of baseline lymphocyte subsets for treatment efficacy in enrolled patient. **(A-F)** The correlation between specific baseline lymphocyte subsets levels and treatment response in enrolled lung cancer patients. **(G-K)** The ROC curves of specific baseline lymphocyte subsets levels and treatment response in enrolled lung cancer patients. * P<0.05, **P<0.01, *** P<0.001, NS, Not significant.

We examined whether pre-treatment levels of peripheral lymphocyte subsets could distinguish patients who would benefit from systemic therapy, including chemotherapy administered alone or in combination with immunotherapy. To this end, we assessed baseline lymphocyte profiles in all 171 enrolled patients and then compared these parameters between those classified as responders (CR, PR) and non-responders (SD, PD). As illustrated in **Figure 1**, the scatter plots illustrate that several lymphocyte subsets differed significantly between the two groups. Patients achieving favorable responses demonstrated higher absolute counts of CD3^-^CD16^+^CD56^+^ cells (*p* = 0.026) and CD3^-^CD19^+^ cells (*p* = 0.0044), as well as an elevated CD4^+^/CD8^+^ ratio (*p* = 0.032) (**Figures 1A, B, F**). Conversely, these individuals exhibited lower baseline levels of CD3^+^ (*p* < 0.001) and CD3^+^CD8^+^ cells (*p* < 0.001) (**Figures 1C, E**). No significant difference was detected in the absolute CD3^+^CD4^+^ cell count between responders and non-responders (**Figure 1D**). Subsequent receiver operating characteristic (ROC) analyses supported the predictive utility of these subsets. Specifically, the baseline frequencies of CD3^-^CD16^+^CD56^+^, CD3^-^CD19^+^, CD3^+^, and CD3^+^CD8^+^ cells, along with the CD4^+^/CD8^+^ ratio, all displayed measurable predictive power, with corresponding AUC values of 0.587, 0.618, 0.679, 0.665, and 0.604, respectively (**Figures 1G-K**).

**Figure 2:**
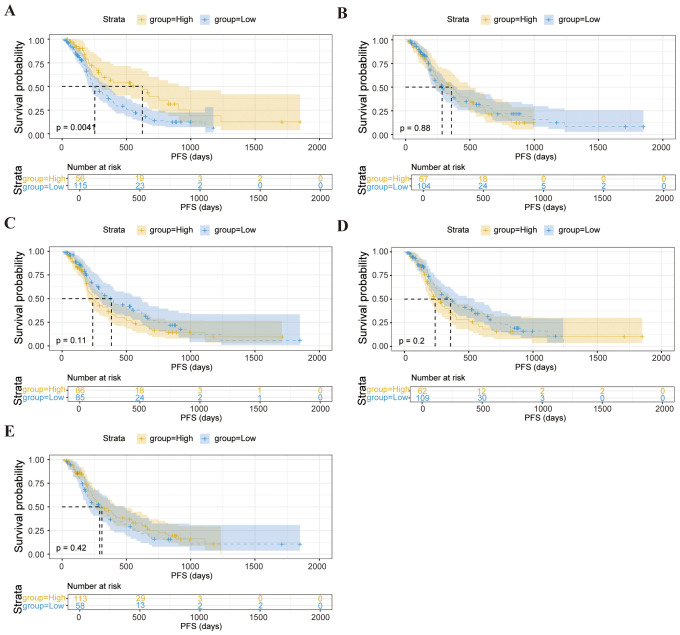
Kaplan-Meier survival curve analysis showing the relationship between values of CD3^-^CD16^+^CD56^+^ cells **(A)**, CD3^-^CD19^+^ **(B)**, CD3^+^ T cells **(C)**, CD3^+^CD8^+^ T cells **(D)**, CD4^+^/CD8^+^ ratio **(E)** and PFS in enrolled patients.

We further explored whether these baseline lymphocyte characteristics could forecast survival outcomes. Notably, elevated counts of CD3^-^CD16^+^CD56^+^ cells were correlated with a significantly prolonged PFS (*p* = 0.0041), as depicted in **Figure 2A**. These findings suggest that pretreatment lymphocyte profiles may serve as valuable biomarkers for anticipating both immediate responses and longer-term therapeutic benefits.

### Predictive value of baseline lymphocyte subsets for therapeutic outcomes in patients receiving chemotherapy combined with immunotherapy

**Figure 3:**
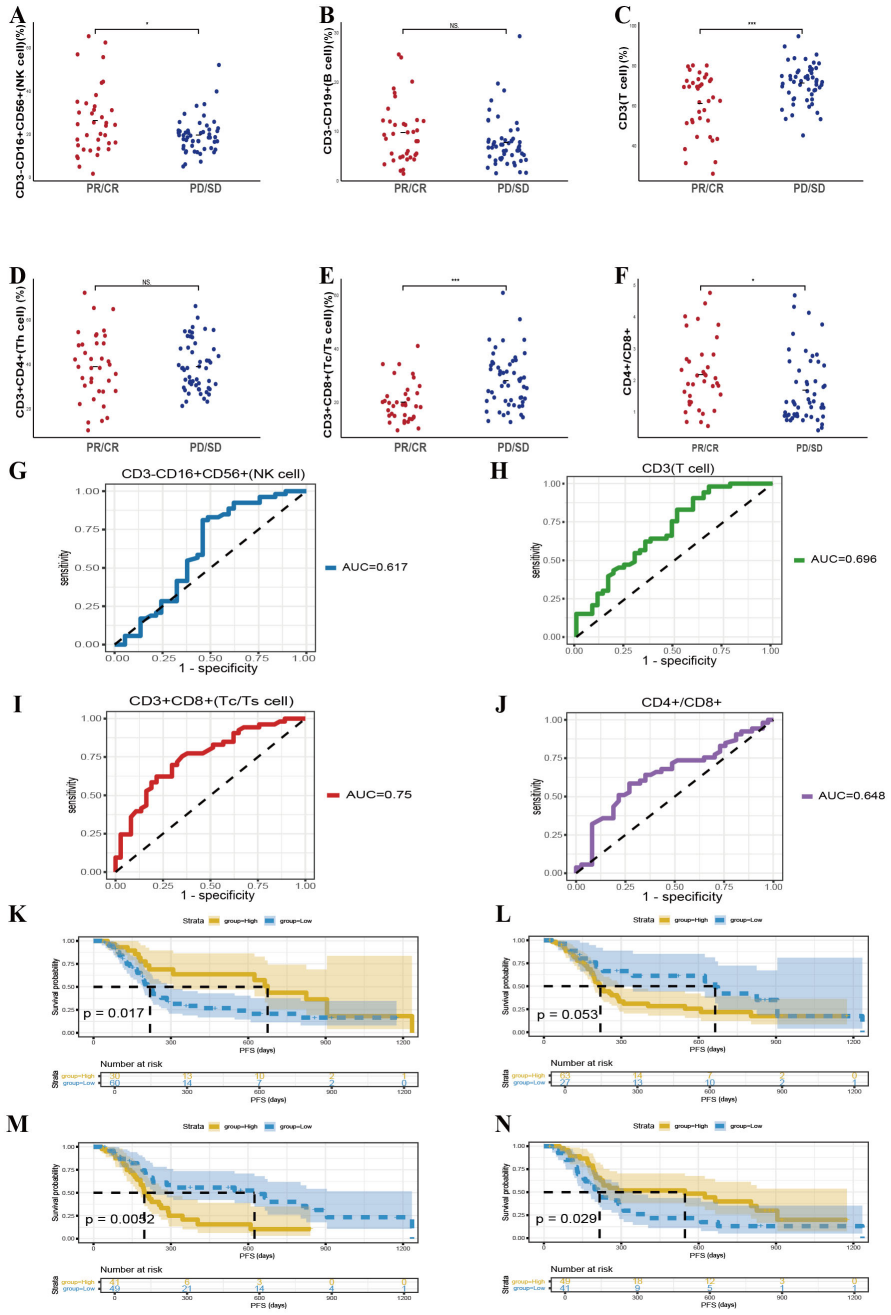
Predictive value of baseline lymphocyte subsets for therapeutic outcomes in patients receiving chemotherapy combined with immunotherapy. **(A-F)** The correlation between specific baseline lymphocyte subsets levels and combination response in lung cancer participants. **(G-J)** The ROC curves of specific baseline lymphocyte subsets levels and combination response in lung cancer participants. Kaplan-Meier survival curve analysis showing the relationship between values of CD3^-^CD16^+^CD56^+^ cells **(K)**, CD3^+^ T cells **(L)**, CD3^+^CD8^+^ T cells **(M)**, CD4^+^/CD8^+^ ratio **(N)** and PFS. NS, Not significant.

Chemotherapy integrated with immunotherapy represents an essential therapeutic approach for patients with locally advanced or metastatic lung cancer. To clarify whether baseline peripheral lymphocyte subset profiles can predict treatment efficacy within this specific regimen, we evaluated 90 patients undergoing combination therapy, of whom 37 exhibited treatment responses. Our analysis revealed that patients demonstrating that elevated baseline counts of CD3^-^CD16^+^CD56^+^ cells (*p* = 0.02) and a higher CD4^+^/CD8^+^ T cell ratio (*p* = 0.037) were linked to increased efficacy of immunotherapy (**Figures 3A, F**). Conversely, lower baseline levels of CD3^+^ T cells (*p* < 0.001) and CD3^+^CD8^+^ T cells (*p* < 0.001) correlated with better therapeutic responses, indicating an inverse relationship between these cell populations and short-term efficacy (**Figures 3C, E**). No other lymphocyte subsets, such as CD3^-^CD19^+^ cells or CD3^+^CD4^+^ cells, were significantly linked to response outcomes. ROC curve analysis supported these findings, showing that pretreatment levels of CD3^-^CD16^+^CD56^+^ cells, CD3^+^ T cells, CD3^+^CD8^+^ T cells, and the CD4^+^/CD8^+^ ratio could serve as predictive indicators for the efficacy of combined therapy, with AUC values of 0.617, 0.696, 0.75, and 0.648, respectively (**Figures 3G-J**).

These baseline lymphocyte features also corresponded with PFS in patients receiving combined modalities. Kaplan–Meier survival analysis demonstrated that elevated CD3^-^CD16^+^CD56^+^ cell counts (*p* = 0.017) and higher CD4^+^/CD8^+^ T-cell ratios (*p* = 0.029) were significantly associated with superior survival outcomes, while lower levels of CD3^+^CD8^+^ T cells (*p* = 0.0052) also predicted improved survival (**Figures 3K-N**). In sum, these data suggest that evaluating specific baseline lymphocyte subsets may help forecast both immediate and longer-term benefits in patients undergoing chemotherapy combined with immunotherapy, thereby providing a valuable tool for therapeutic decision-making and optimization.

### Predictive value of baseline lymphocyte subsets on the efficacy of chemotherapy in patients

**Figure 4:**
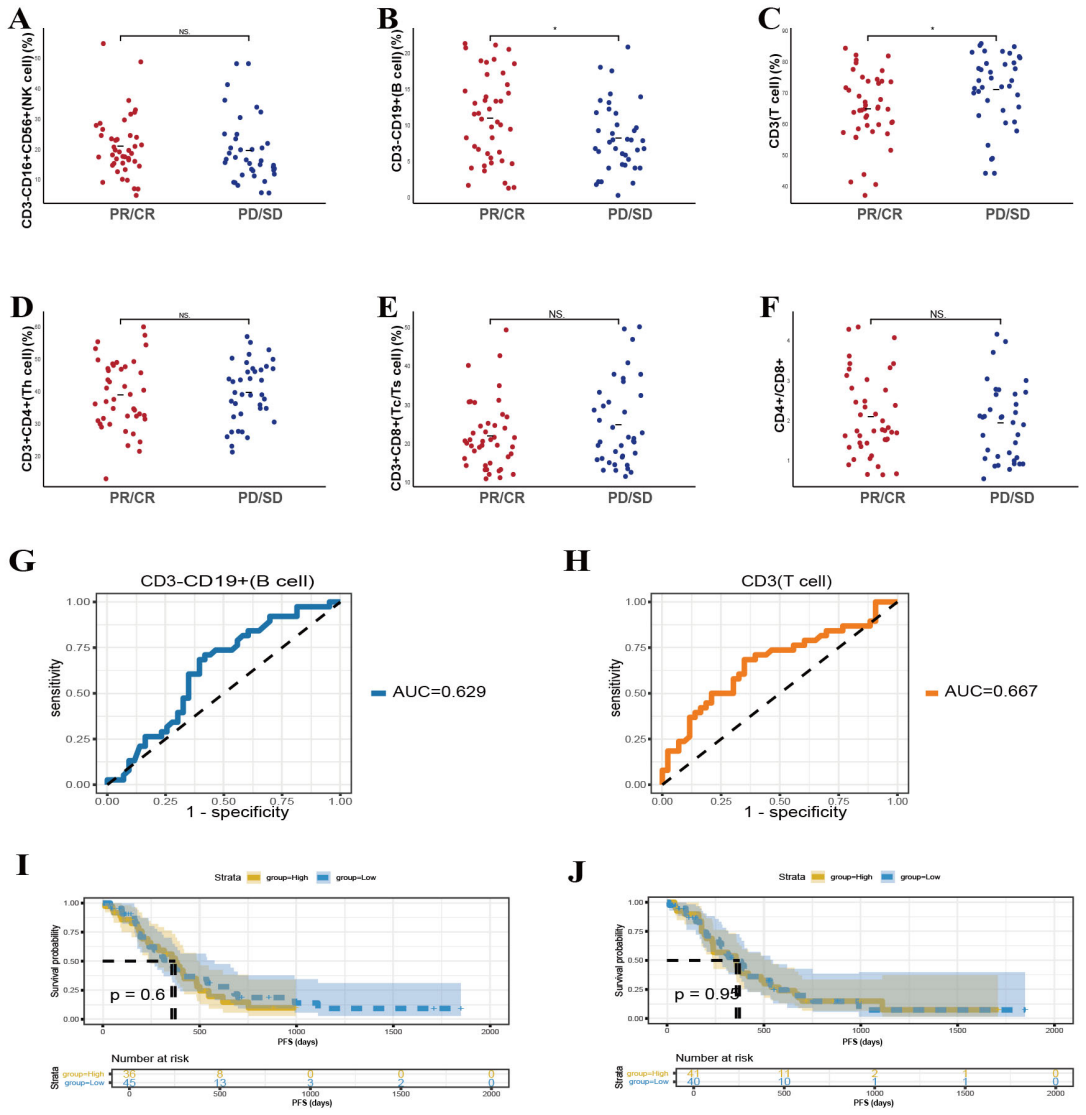
Predictive value of baseline lymphocyte subsets on the efficacy of chemotherapy in patients. **(A-F)** The correlation between specific baseline lymphocyte subsets levels and chemotherapy response in lung cancer participants. **(G, H)** The ROC curves of specific baseline lymphocyte subsets levels and chemotherapy response in lung cancer participants. Kaplan-Meier survival curve analysis showing the relationship between values of CD3^-^CD19^+^ cells **(I)**, CD3^+^ T cells **(J)**. * P<0.05, NS, Not significant.

In addition to exploring patients treated with combined chemotherapy and immunotherapy, we further evaluated whether baseline peripheral lymphocyte subsets could predict treatment responses in individuals receiving chemotherapy alone. This analysis aimed to determine the extent to which these immune parameters could serve as independent indicators of clinical benefit in the absence of immunotherapy. In the cohort receiving chemotherapy alone, elevated baseline CD3^-^CD19^+^ T cell counts (**Figure 4B**, *p* = 0.026) and reduced CD3^+^ T cell counts (**Figure 4C**, *p* = 0.022) were significantly associated with improved response rates. In contrast, no statistically meaningful connections were identified between other examined lymphocyte subsets, including CD3^-^CD16^+^CD56^+^, CD3^+^CD4^+^, CD3^+^CD8^+^ T cells, or the CD4^+^/CD8^+^ ratio and chemotherapy outcomes. These observations imply that chemotherapy may predominantly influence innate immune responses, while exerting comparatively limited effects on adaptive immunity.

ROC curve analyses further supported the predictive potential of CD3^-^CD19^+^ T-cell and CD3^+^ T cell counts, with AUC values of 0.629 and 0.667 (**Figures 4G, H**), respectively. Nevertheless, subsequent survival assessments revealed no significant associations between these particular baseline lymphocyte parameters and long-term survival, as depicted in **Figures 4I, J**. By examining the pretreatment lymphocyte profiles, we assessed their correlation with therapeutic outcomes and PFS. This evaluation allowed us to discern whether similar patterns observed in combination therapy cohorts persisted in patients managed solely with chemotherapy, thus identifying a broader applicability of these biomarkers as predictors of clinical efficacy.

### Predictive value of second cycle lymphocyte subsets on the therapeutic efficacy

**Figure 5:**
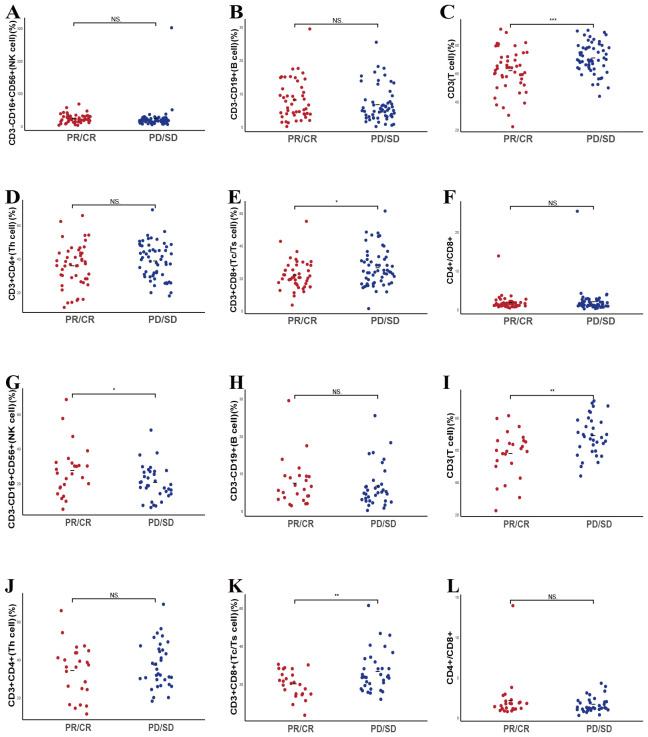
Predictive value of second cycle lymphocyte subsets on the therapeutic efficacy. **(A-F)** The correlation between specific post-treatment lymphocyte subsets levels and treatment response in enrolled lung cancer participants. **(G-L)** The correlation between specific baseline lymphocyte subsets levels and chemo-immunotherapy combination response in lung cancer participants. NS, Not significant.

We further explored whether alterations in lymphocyte subsets over two treatment cycles could forecast therapeutic responses among patients undergoing chemotherapy alone or chemotherapy combined with immunotherapy. A total of 104 patients provided longitudinal lymphocyte subset data spanning two consecutive cycles. Evaluation of these second-cycle values revealed that lower CD3^+^ (*p* < 0.001) and CD3^+^CD8^+^ T cell counts (*p* = 0.023) remained positively associated with favorable responses, consistent with our baseline observations (**Figures 5C, E**). No other subsets displayed significant relationships with therapeutic efficacy at this time point (**Figures 5A, B, D, F**).

When focusing on the 59 patients receiving combined chemotherapy and immunotherapy, we noted that higher CD3^-^CD16^+^CD56^+^ cell counts (*p* = 0.05) and lower CD3^+^ (*p* = 0.003) and CD3^+^CD8^+^ T cell levels (*p* = 0.0061) in the second cycle correlated with improved outcomes, mirroring the baseline trends (**Figures 5G, I, K**). Interestingly, the CD4^+^/CD8^+^ T cell ratio, which at baseline had demonstrated predictive value, did not show a significant second-cycle association with treatment efficacy (**Figures 5H, J, L**). Among the 45 patients treated exclusively with chemotherapy, only reduced CD3^+^ T cell counts predicted a better response, and no significant correlations emerged for other subsets (**Supplementary Figure S2**).

### Dynamic changes in lymphocyte subsets between responders and non-responders

To further elucidate the link between dynamic peripheral lymphocyte fluctuations and treatment efficacy, we assessed changes in lymphocyte subsets across two successive treatment cycles in both responder and non-responder groups. Data were collected at baseline and following treatment initiation. Among the 59 patients treated with chemotherapy or combination immunotherapy, 34 achieved a response, while 25 did not. Analysis of pre- and post-treatment values did not reveal significant shifts in any lymphocyte subset within either group (**Supplementary Figure S3**). Similarly, in the 45 patients receiving combination immunotherapy (23 responders, 22 non-responders), no significant alterations were detected (**Supplementary Figure S4**).

Additionally, to further investigate the relationship between disease progression and lymphocyte subsets, we innovatively examined baseline lymphocyte profiles and their levels at the time of disease progression in both responder and non-responder cohorts. A total of 41 patients recorded lymphocyte profiles in both baseline and disease progression, among them 17 patients were in responder cohort, while 24 in non-responder cohort. Comparative analysis revealed no significant differences in lymphocyte subsets between these two time points in both two cohorts, indicating that these immune parameters remained relatively stable throughout the disease course, irrespective of treatment outcomes (**Supplementary Figure S5**).

### Construction of nomogram models for predicting efficacy

**Figure 6:**
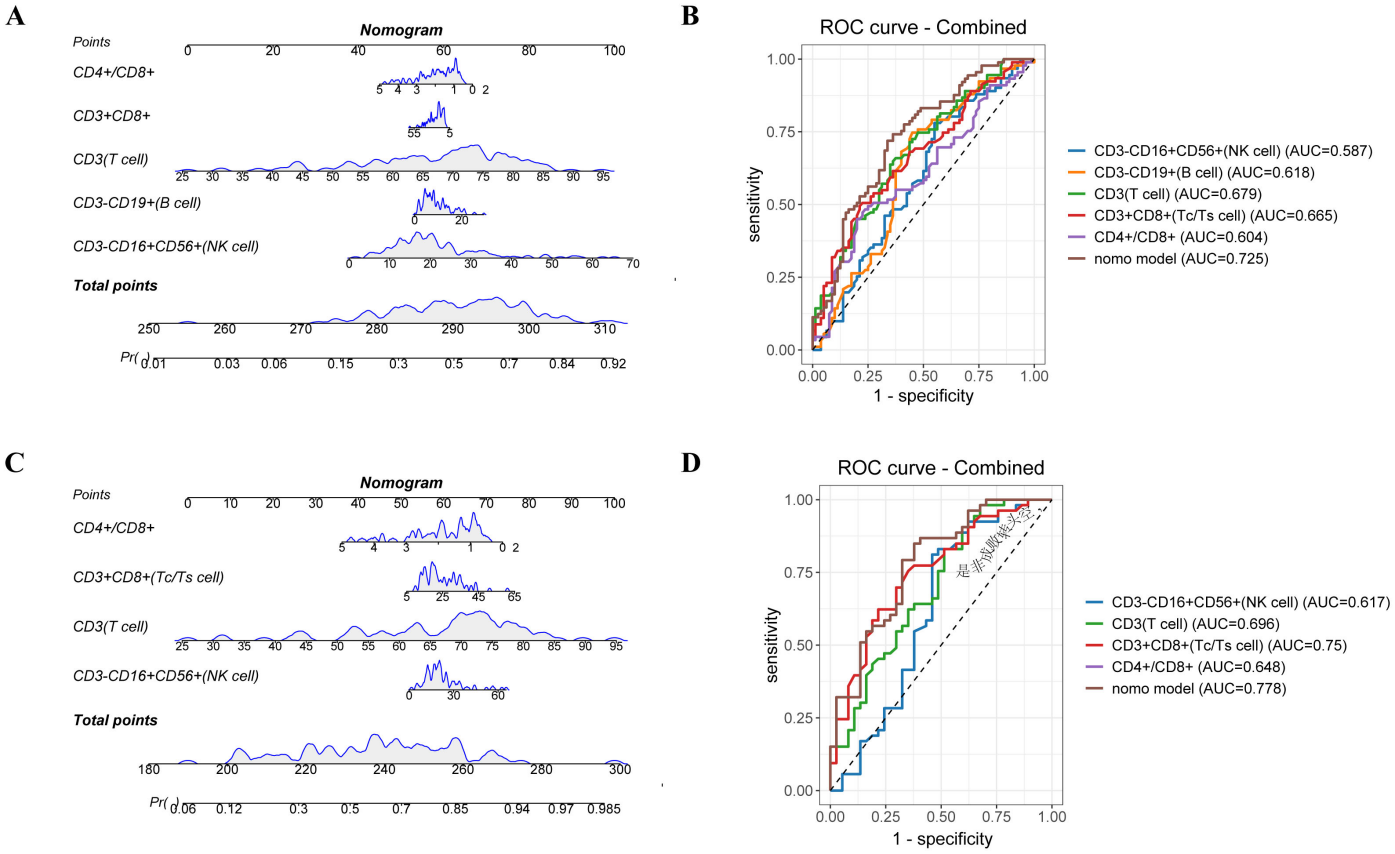
Nomogram model predicts treatment efficacy of lymphocyte subsets between the response and non-response groups. A nomogram predicting the value of lymphocyte subsets and treatment efficacy for enrolled patients. **(B)** The ROC curves of nomogram and different risk factors for enrolled patients. **(C)** A nomogram predicting the value of lymphocyte subsets and treatment efficacy for patients with chemo-immunotherapy combination treatment. **(D)** The ROC curves of nomogram and different risk factors for patients with chemo-immunotherapy combination treatment.

To further explore, we included all variables identified as significant in the univariate logistic regression analysis (**Supplementary Tables S1**, **S2**). Leveraging these key predictors, we constructed two nomograms to estimate response likelihood (**Figure 6**).

In the entire cohort, the nomogram integrated five parameters—CD3^-^CD16^+^CD56^+^, CD3^-^CD19^+^, CD3^+^, CD3^+^CD8^+^ T cells, and the CD4^+^/CD8^+^ T cell ratio. The AUC-ROC demonstrated discriminative ability with an AUC of 0.778, surpassing the predictive value of any individual factor (**Figures 6A, B**). The calibration curve indicated good agreement between predicted and observed probabilities, with a mean absolute error (MAE) < 0.05, confirming the reliability of the model.

In the subgroup receiving chemo-immunotherapy, a similar nomogram incorporating four variables—CD3^-^CD16^+^CD56^+^, CD3^+^, CD3^+^CD8^+^T cells, and the CD4^+^/CD8^+^ ratio—achieved an AUC of 0.725 (**Figures 6C, D**). The model effectively stratified patients into high and low-risk groups, with a statistically significant separation in PFS (P = 0.029) (**Supplementary Figure S6**), further supporting its utility in predicting immunotherapy outcomes. Calibration analyses showed excellent consistency between predicted and actual responses, reinforcing the robustness of the model in this subset (**Supplementary Figure S7**).

However, in the chemotherapy-only subgroup, the nomogram model demonstrated limited predictive capability. Notably, the model integrating both CD3^+^ and CD3^-^CD19^+^ cell subsets yielded similar predictive accuracy to the CD3^+^ subset alone, with AUC values of 0.66, indicating that the addition of B cell parameters did not enhance prognostic discrimination in chemotherapy-treated patients (**Supplementary Figure S8**). These findings suggest that chemotherapy-treated patients, peripheral lymphocyte subsets alone may not fully capture the determinants of treatment response.

## Discussion

Immunotherapy has markedly transformed the treatment landscape for locally advanced and metastatic lung cancer, yet a subset of patients experiences limited or transient benefits (18, 19). Identifying reliable predictive biomarkers is crucial to refining patient selection and optimizing therapeutic strategies. While tumor tissue–based biomarkers have received considerable attention, challenges related to acquisition and representation limit their practical utility. By contrast, peripheral blood offers a more accessible sample source and may reflect the systemic immune milieu in a manner that complements localized tumor information (20–22). In this condition, assessing peripheral blood lymphocyte subsets, which are integral to both innate and adaptive immune responses, holds promise as a noninvasive method for gauging the likelihood of a favorable outcome.

Lymphocytes are integral to immune surveillance and tumor control (16). The three major subsets, T, B, and NK cells, are the primary effector of the adaptive immune system (23). T cells, identifiable by the CD3 surface marker, differentiate into CD4^+^ and CD8^+^ T cells upon antigen recognition, both of which are crucial for antitumor immunity. Previous studies have demonstrated that patients responding to immunotherapy often exhibit reduced baseline levels of circulating T cells compared to non-responders (24). CD4^+^ T cells, as the main helper cells, enhance B cell antibody production and regulate immune responses mediated by other T cells. Recent evidence suggests that the CD4^+^ T cell subset plays a protective role against cancer progression by augmenting the tumoricidal activity of other antitumor effector cell subsets (25). However, CD8^+^ T cells are functionally heterogeneous (26). Differences in the expression levels of molecular markers linked to T cell exhaustion (PD-1, CTLA-4, CD160) and T cell senescence (CD57, absence of CD28) in CD8^+^ T cells may influence the efficacy of immunotherapy in distinct ways (27). Notably, CD8^+^ T cells can be further subdivided based on CD28 expression, which modulates the immune response either positively or negatively (28). An increase in CD8^+^ CD28− T cells is associated with impaired immune function (26). Maintaining a homeostatic balance between these subsets is essential for a robust immune response. In our study, higher circulating levels of total CD3^+^CD8^+^ T cells were unexpectedly associated with poorer short-term treatment outcomes. This may be attributed to an increased proportion of dysfunctional or non-tumor-reactive CD8^+^ subsets, such as CD8^+^CD28^−^ cells, which are linked to impaired immune function. Previous studies have shown that moderately elevated CD8^+^CD28^+^ T cell counts may predict favorable responses, while excessive elevations may increase the risk of immune-related adverse events (irAEs), ultimately compromising therapeutic benefit. Given that without further functional stratification, the observed association likely reflects expansion of ineffective or exhausted CD8^+^ T cell populations rather than active cytotoxic immunity. These findings highlight the importance of incorporating functional profiling—such as immune checkpoint expression and antigen specificity—into future analyses. Furthermore, A decreased CD4^+^/CD8^+^ ratio often signifies an immunosuppressive state, commonly observed in cancer patients, where compromised cellular immune function diminishes the body’s ability to recognize and eliminate malignant cells (29).

B cells, characterized by the CD19 surface marker, primarily secrete antibodies against tumor-associated antigens and coactivate CD8^+^ T cells in conjunction with CD4^+^ T cells (30, 31). NK cells, identified by the CD56 marker, constitute the first line of defense against tumors and pathogens (32). The cytotoxic and immunomodulatory effects of them are critical to the overall status of the tumor microenvironment (33). Current research is actively exploring NK cell–based immunotherapies as a complementary strategy to enhance antitumor responses (32, 34–36). Various factors released from tumor cells facilitate the activation and proliferation of lymphocytes, thereby contributing to tumor eradication during cancer treatment, particularly immunotherapy (37, 38). However, the presence of large numbers of inactive or “bystander” lymphocytes within the tumor immune microenvironment can undermine therapeutic efficacy (39). Consequently, assessing specific lymphocyte subsets may not fully capture the actual immune capacity, highlighting the need for comprehensive immune profiling (40).

In this study, we systematically analyzed the predictive value of peripheral blood lymphocyte subsets across different treatment modalities. Our findings indicate that specific lymphocyte subsets measured before and shortly after treatment initiation hold significant potential in identifying patients who are more likely to benefit from these treatments while the combination of several lymphocyte subsets can be more crucial. These insights contribute to the growing body of evidence supporting the use of peripheral blood immune profiling as a valuable tool in personalizing cancer immunotherapy.

By analyzing the baseline and early-treatment peripheral blood lymphocyte profiles in patients with advanced lung cancer receiving chemotherapy alone or in combination with immunotherapy, one key finding was that distinct subsets at baseline, including CD3^-^CD16^+^CD56^+^ cells, CD3^-^CD19^+^ cells, and an increased CD4^+^/CD8^+^ T cell ratio, were more frequently associated with favorable responses. In contrast, higher levels of CD3^+^ and CD3^+^CD8^+^ T cells tended to correlate with poorer short-term efficacy. These patterns persisted after additional treatment cycles, particularly in those receiving immunotherapy-containing regimens, underscoring the relevance of these immune parameters as early predictors.

Our study further highlights differences based on treatment modalities. In patients who received immunotherapy-containing regimens, baseline elevations in CD3^-^CD16^+^CD56^+^ cells and a higher CD4^+^/CD8^+^ ratio were linked to improved outcomes. Conversely, in individuals treated with chemotherapy alone, only certain subsets, such as CD3^-^CD19^+^ cells and lower CD3^+^ T-cell counts, showed predictive value for short-term responses. Although these associations did not translate into significant survival advantages for the chemotherapy-only group, they still indicate that early immune subsets could help refine patient selection and timing of treatment adjustments. Meanwhile, we innovatively examined the link between dynamic peripheral lymphocyte fluctuations and treatment efficacy, especially the relationship between disease progression and lymphocyte subsets. However, the dynamic assessments of peripheral blood lymphocyte subsets did not reveal substantial shifts correlated with long-term efficacy once the disease progressed, suggesting that these biomarkers might be more relevant at the initiation and early stages of therapy. While the absence of significant alterations at progression may reflect complex host-tumor interactions that evolve over time, it underscores the importance of evaluating immune parameters before or shortly after therapy begins, rather than later in the disease course.

Based on the identified predictive factors, we developed a nomogram model that integrated multiple lymphocyte subsets. This model demonstrated robust discrimination (AUC = 0.778 in the entire cohort) and calibration, outperforming single biomarkers. The model was particularly effective in patients receiving immunotherapy-containing regimens (AUC = 0.725), underscoring the relevance of systemic immune profiling in predicting response to immune checkpoint inhibitors. These findings support the integration of lymphocyte subset analysis into clinical decision-making to refine patient selection and therapeutic strategies. By enhancing predictive capacity, such multifactorial tools could support personalized treatment decisions, allowing clinicians to identify patients most likely to benefit from immunotherapy-containing regimens and consider early therapeutic modifications if immune parameters suggest limited gains.

Interestingly, in the chemotherapy-only cohort, the nomogram model performed poorly, with no substantial advantage of integrating B cells over CD3^+^ T cells alone. Several factors may explain this finding. Unlike immunotherapy, which relies on immune activation, chemotherapy exerts direct cytotoxic effects, making lymphocyte profiles less reflective of response. Limited role of B cells in chemotherapy: While B cells contribute to immunotherapy efficacy through antigen presentation and antibody production, their role in chemotherapy response is less defined. And also, the lack of strong predictive power suggests that chemotherapy response may be influenced by tumor-intrinsic factors or inflammatory markers beyond peripheral lymphocyte subsets. Future studies should explore the integration of inflammatory markers (e.g., CRP, IL-6), tumor-intrinsic features, or functional immune assays to enhance prediction accuracy in chemotherapy-treated patients.

Our findings demonstrated that peripheral blood lymphocyte subsets could serve as dynamic, non-invasive biomarkers for assessing treatment efficacy in patients with lung cancer. The nomogram we developed provides a reliable, individualized approach to predicting treatment outcomes, particularly in patients receiving chemotherapy and immunotherapy. This predictive model can be integrated into routine oncology practice to stratify patients and guide treatment decisions. Specifically, patients with higher immune activation may benefit from combination therapies, while those with lower immune responses may require alternative strategies or more intensive monitoring. Additionally, the non-invasive nature of peripheral blood tests allows for frequent and dynamic monitoring, enabling clinicians to adjust regimens in real time based on evolving immune profiles. Given the promising potential of this model, we believe it could guide early therapeutic modifications.

Despite these encouraging findings, certain limitations warrant consideration. First, this study was conducted at a single center, necessitating further validation in multicenter, prospective cohorts to enhance the generalizability of our findings. Second, we primarily analyzed baseline peripheral blood lymphocyte subsets, whereas functional immune assessments, including detailed analyses of T-cell subsets (such as regulatory T cells, Th17 cells) and tumor antigen-specific T or NK cells, were not included, limiting our understanding of the functional dynamics underpinning immune responses. Third, while PD-L1 and TMB are recognized biomarkers for predicting immunotherapy outcomes, this study specifically focused on peripheral blood lymphocyte subsets as a complementary and non-invasive predictive tool. Future studies integrating these established indicators, including PD-L1 and TMB, may enhance the predictive performance. Furthermore, the divergent roles of B cells in chemotherapy versus immunotherapy remain to be elucidated, particularly through high-resolution techniques such as single-cell RNA sequencing to delineate functional heterogeneity within B cells. Lastly, we focused on peripheral blood, which may not mirror the full complexity of the tumor microenvironment. Future investigations should incorporate tissue-based correlatives and spatial immune profiling to gain a more holistic understanding of tumor–immune interactions.

To further advance this line of research, future studies should explore dynamic immune monitoring strategies that integrate peripheral and tumor-infiltrating immune components across multiple treatment stages. Incorporating machine learning or artificial intelligence-based modeling may enhance the predictive accuracy of nomogram tools by capturing nonlinear interactions among immune variables. Moreover, functional characterization of circulating lymphocyte subsets—such as cytotoxicity assays or immune checkpoint expression analysis—could provide mechanistic insights into their role in modulating treatment response. From a translational perspective, the peripheral immune signature identified in this study has the potential to guide real-time treatment stratification, helping clinicians identify patients more likely to benefit from immunotherapy or combination regimens. Ultimately, this may enable more personalized, adaptive treatment strategies that improve clinical outcomes while minimizing unnecessary toxicity and cost.

Overall, by emphasizing individualized therapy, early intervention, and dynamic monitoring, the proposed nomogram-based strategy could enhance patient outcomes and resource allocation in advanced lung cancer management. Nonetheless, larger and more heterogeneous validation studies remain essential to confirm its broader clinical utility.

## Conclusion

In conclusion, our study proposes that selected baseline and early-treatment peripheral lymphocyte characteristics may serve as noninvasive predictive indicators of therapeutic efficacy in advanced lung cancer. Moreover, a composite predictive framework incorporating multiple lymphocyte subset parameters can more reliably forecast treatment outcomes compared to reliance on a single biomarker. Integrating these immune parameters into clinical practice could inform more personalized treatment strategies, guide early therapeutic modifications, and ultimately improve patient outcomes. Future research should focus on validating these findings in larger, multi-institutional cohorts and refining nomogram models to include additional biological and clinical factors.

## Data Availability

The raw data supporting the conclusions of this article will be made available by the authors, without undue reservation.

## Supplementary material

Supplementary Figure 1 Flowchart of the study.

Supplementary Figure 2 The correlation between specific post-treatment lymphocyte subsets levels and chemotherapy response in chemotherapy-only patients **(A-F)**.

Supplementary Figure 3 Dynamic changes of lymphocyte subsets Between responders and non-responders in patients with chemotherapy or combination immunotherapy. **(A-F)** The correlation between changes of specific lymphocyte subsets levels across two successive treatment cycles in responder groups. **(G-L)** The correlation between changes of specific lymphocyte subsets levels across two successive treatment cycles in non-responder groups.

Supplementary Figure 4 Dynamic changes of lymphocyte subsets between responders and non-responders in patients with combination treatment. **(A-F)** The correlation between changes of specific lymphocyte subsets levels across two successive treatment cycles in responder groups. **(G-L)** The correlation between changes of specific lymphocyte subsets levels across two successive treatment cycles in non-responder groups.

Supplementary Figure 5 Different time point of lymphocyte subsets between responders and non-responders in lung cancer patients. **(A-F)** Association between specific lymphocyte subsets at baseline and disease progression in responder groups. **(G-L)** Association between specific lymphocyte subsets at baseline and disease progression in non-responder groups.

Supplementary Figure 6 Kaplan-Meier Curves for PFS Based on Nomogram-Derived Risk Stratification. **(A)** Kaplan-Meier curves for PFS in the entire cohort, stratified by nomogram derived risk groups. **(B)** Kaplan-Meier curves for PFS in the chemo-immunotherapy cohort, stratified by nomogram derived risk groups.

Supplementary Figure 7 Decision curve analysis and calibration curves for nomogram model. **(A, B)** DCA and Calibration curves for the nomogram model in the entire cohort. **(C, D)** DCA and Calibration curves for the nomogram model in the chemo-immunotherapy cohort.

Supplementary Figure 8 Predictive performance of the nomogram model for chemotherapy-only patients. **(A)** Nomogram predicting treatment efficacy based on lymphocyte subsets in chemo-only patients. **(B)** The ROC curves comparing the predictive accuracy of the Nomogram model and individual risk factors. **(C, D)** DCA and Calibration curves of nomogram for the established nomogram. **(E)** Kaplan-Meier curves for PFS based on nomogram derived risk stratification.

Supplementary Figure 9 Graphic abstract.

Supplementary Table 1
**(A)** Univariate Logistic regression of lymphocyte subsets for treatment efficacy in enrolled patients. **(B)** Multivariate Logistic regression of lymphocyte subsets for treatment efficacy in enrolled patients.

Supplementary Table 2
**(A)** Univariate Logistic regression of lymphocyte subsets for treatment efficacy in combination patients. **(B)** Multivariate Logistic regression of lymphocyte subsets for treatment efficacy in combination patients.
