# Supplementary figures and images for: The application of peripheral blood immune profiling in personalized treatment of locally advanced and advanced lung cancer: a nomogram approach
Source: Front Oncol. 2025 Sep 1;15:1642829. doi: 10.3389/fonc.2025.1642829 (PMC12433846; doi:10.3389/fonc.2025.1642829)

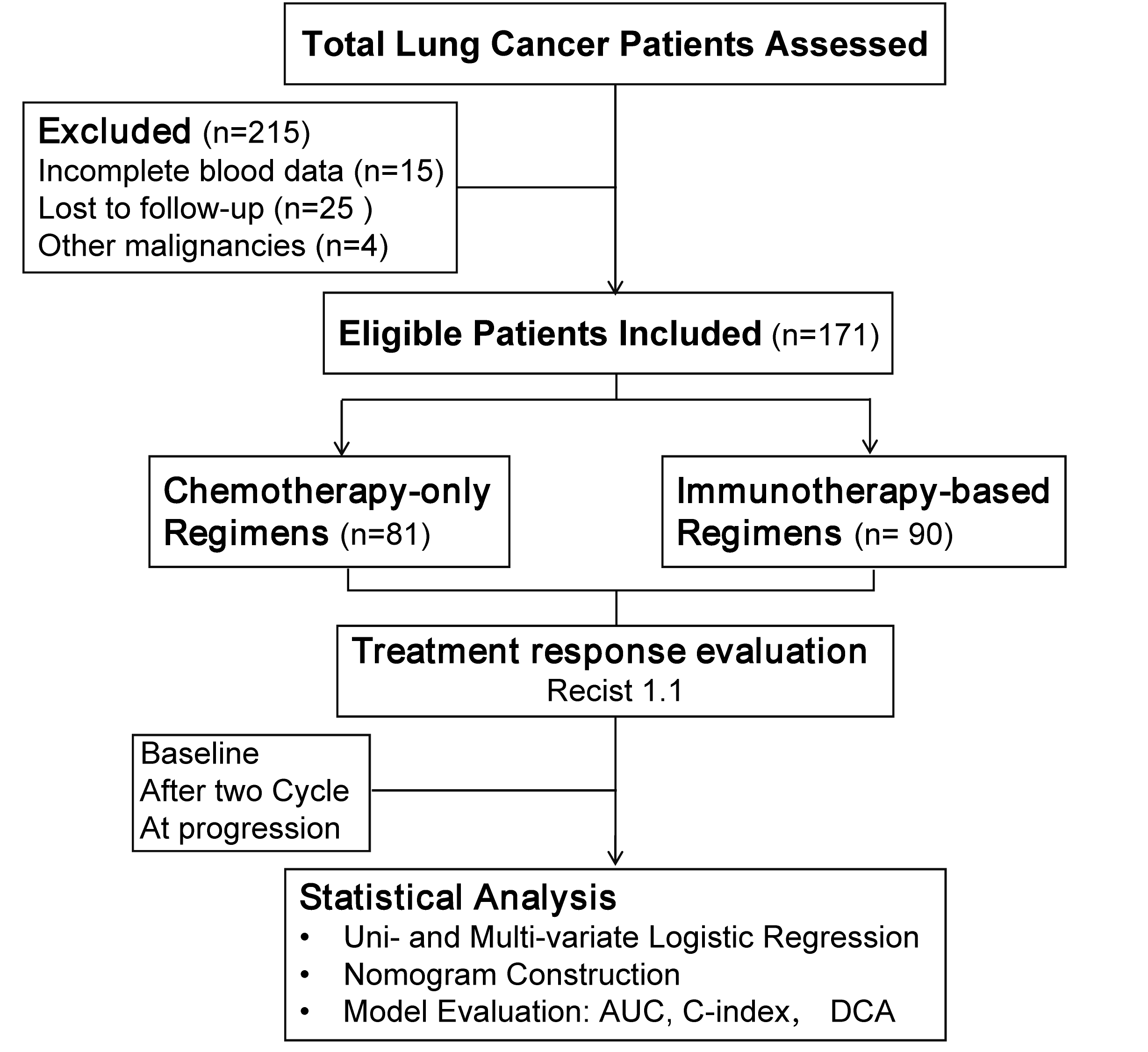

Supplement: Supplementary Figure 1 — Flowchart of the study. [file Image1.tif]

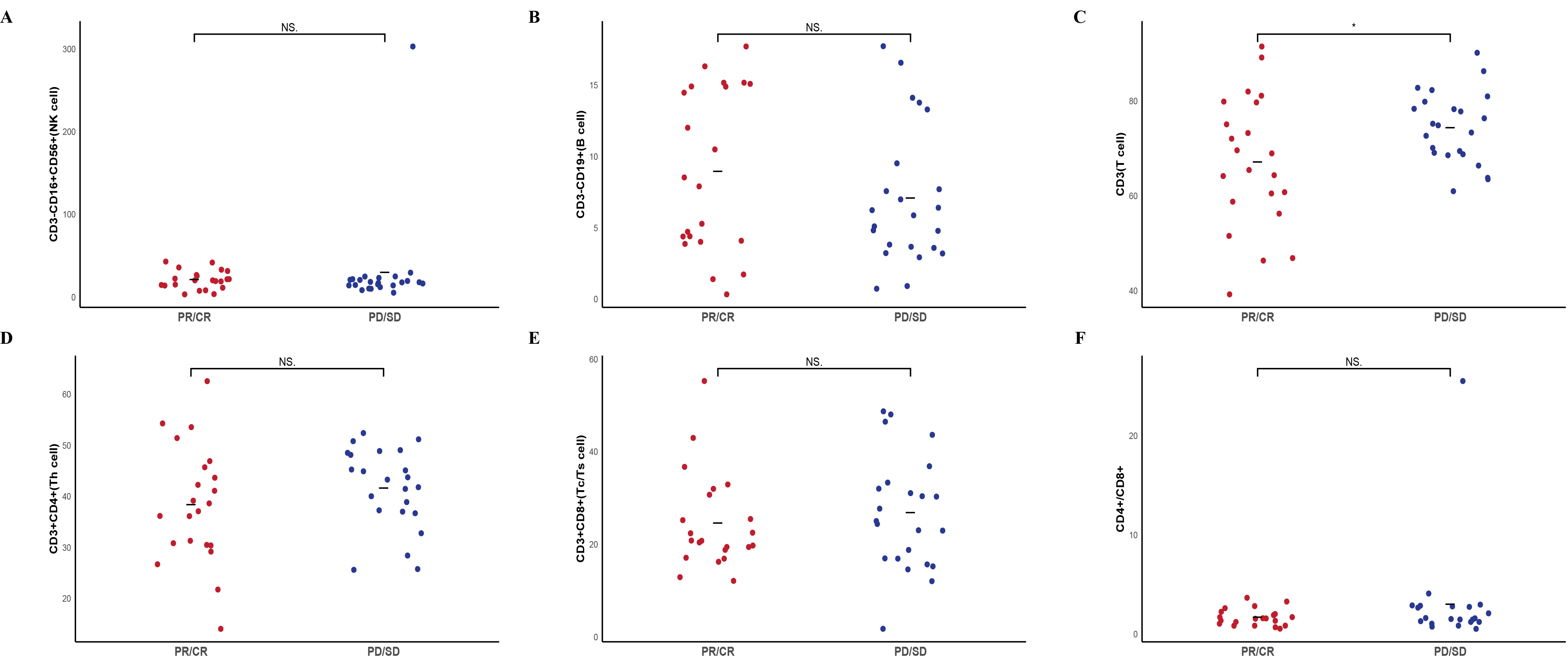

Supplement: Supplementary Figure 2 — The correlation between specific post-treatment lymphocyte subsets levels and chemotherapy response in chemotherapy-only patients (A-F). [file Image2.tif]

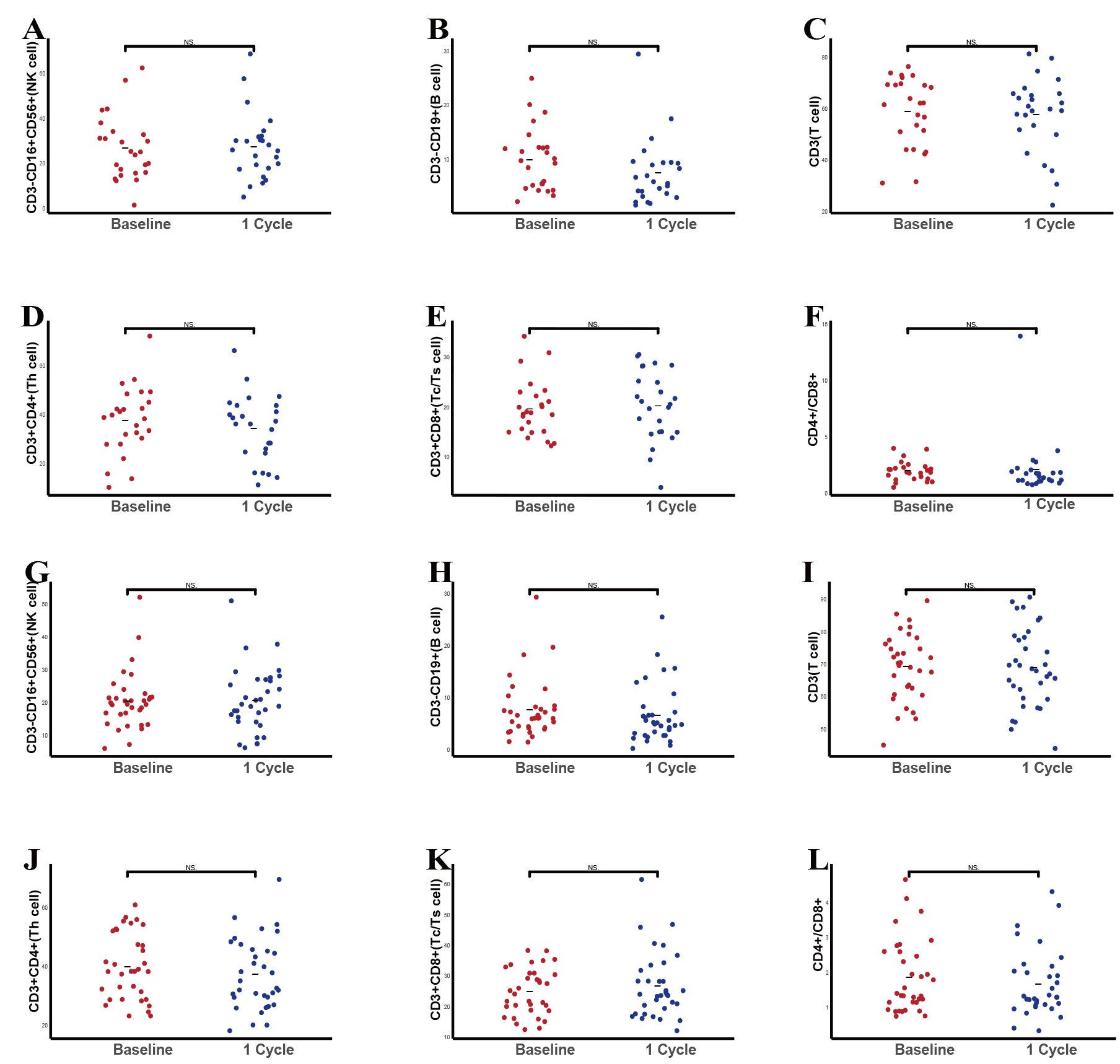

Supplement: Supplementary Figure 3 — Dynamic changes of lymphocyte subsets Between responders and non-responders in patients with chemotherapy or combination immunotherapy. (A-F) The correlation between changes of specific lymphocyte subsets levels across two successive treatment cycles in responder groups. (G-L) The correlation between changes of specific lymphocyte subsets levels across two successive treatment cycles in non-responder groups. [file Image3.tif]

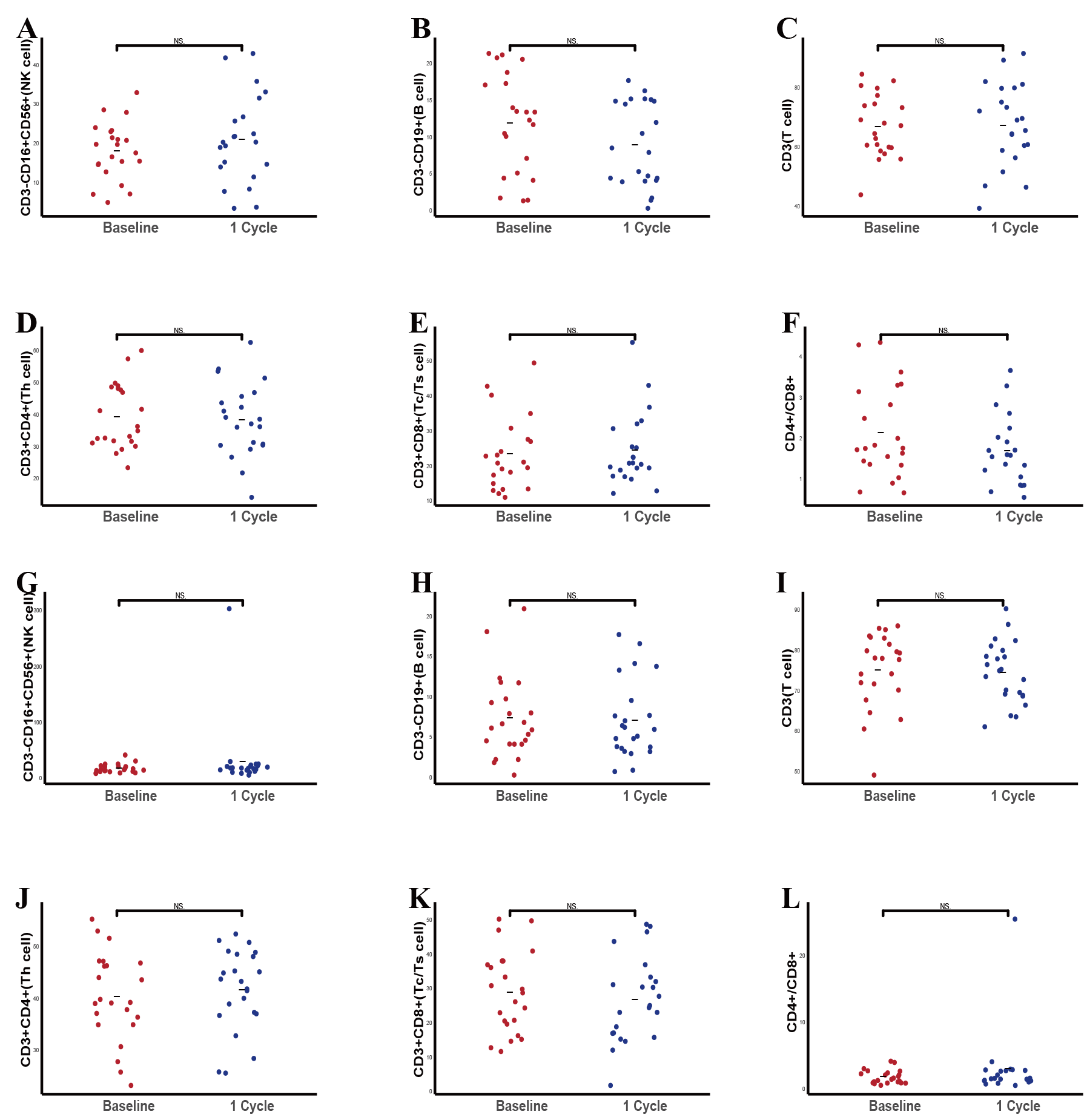

Supplement: Supplementary Figure 4 — Dynamic changes of lymphocyte subsets between responders and non-responders in patients with combination treatment. (A-F) The correlation between changes of specific lymphocyte subsets levels across two successive treatment cycles in responder groups. (G-L) The correlation between changes of specific lymphocyte subsets levels across two successive treatment cycles in non-responder groups. [file Image4.tif]

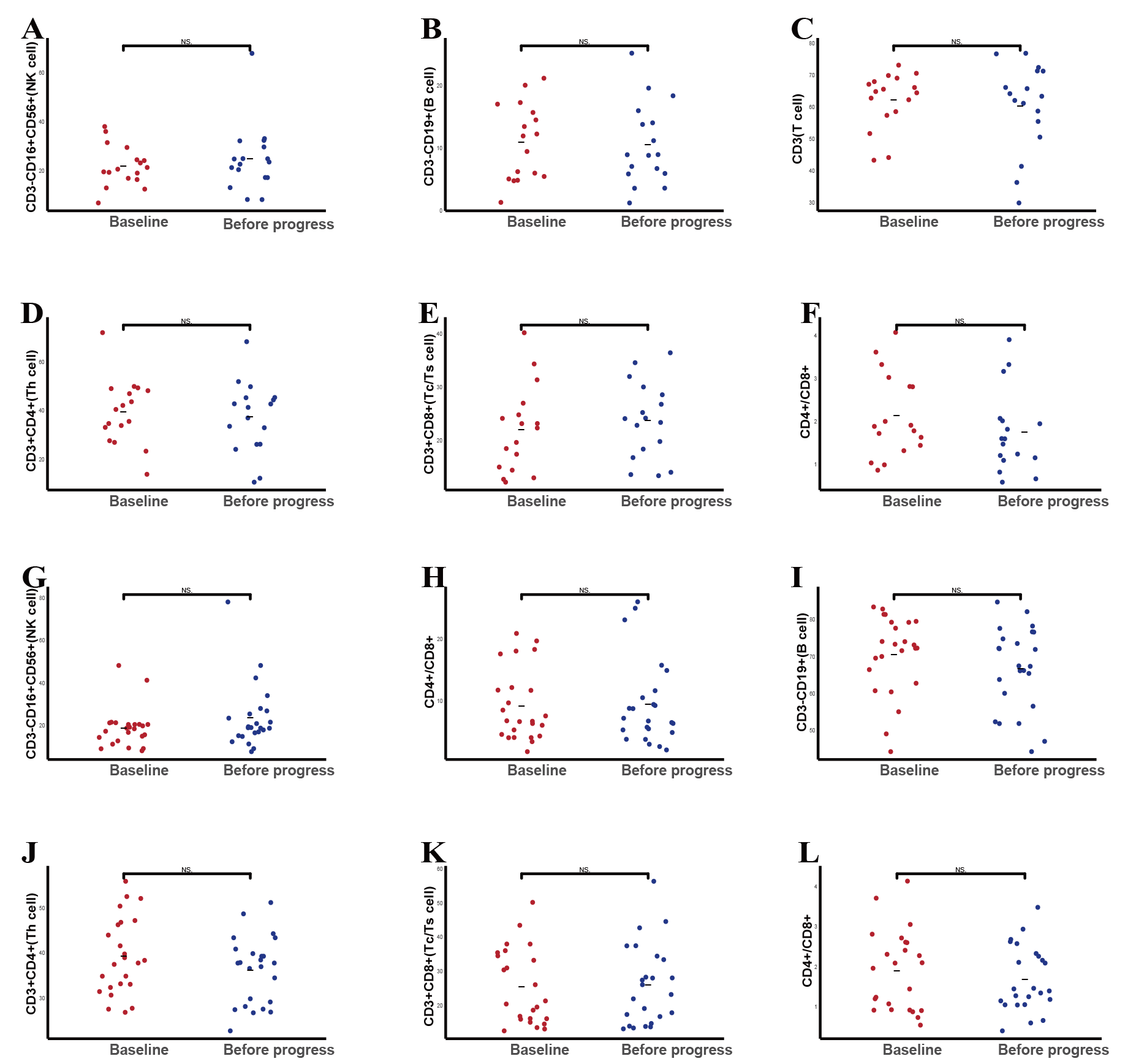

Supplement: Supplementary Figure 5 — Different time point of lymphocyte subsets between responders and non-responders in lung cancer patients. (A-F) Association between specific lymphocyte subsets at baseline and disease progression in responder groups. (G-L) Association between specific lymphocyte subsets at baseline and disease progression in non-responder groups. [file Image5.tif]

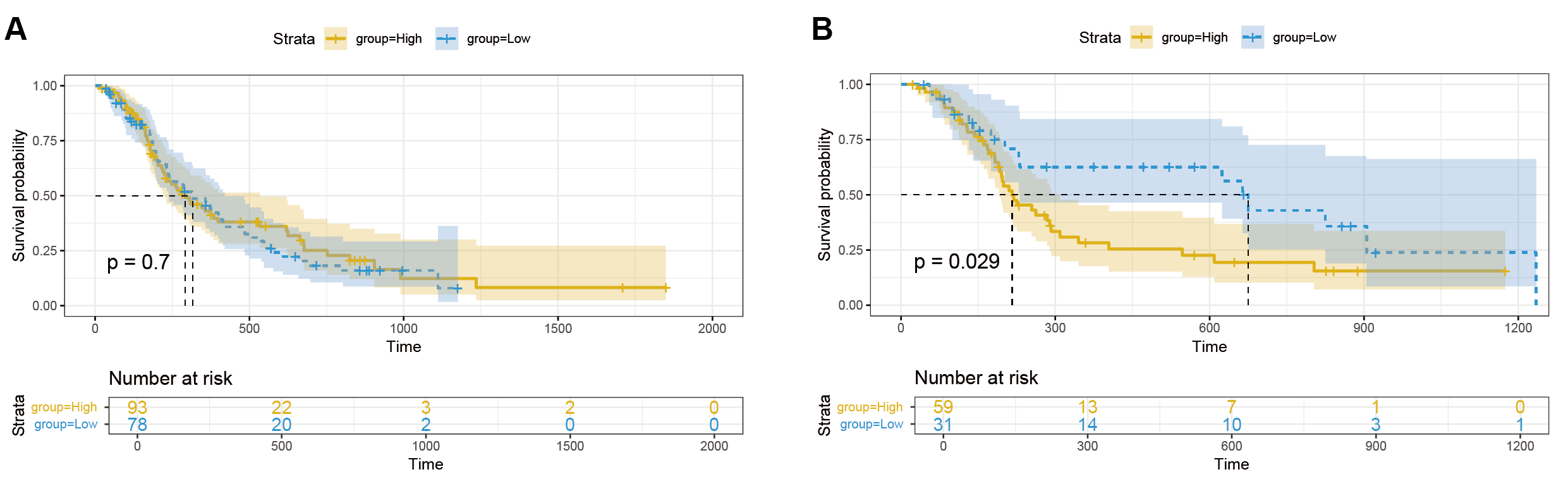

Supplement: Supplementary Figure 6 — Kaplan-Meier Curves for PFS Based on Nomogram-Derived Risk Stratification. (A) Kaplan-Meier curves for PFS in the entire cohort, stratified by nomogram derived risk groups. (B) Kaplan-Meier curves for PFS in the chemo-immunotherapy cohort, stratified by nomogram derived risk groups. [file Image6.png]

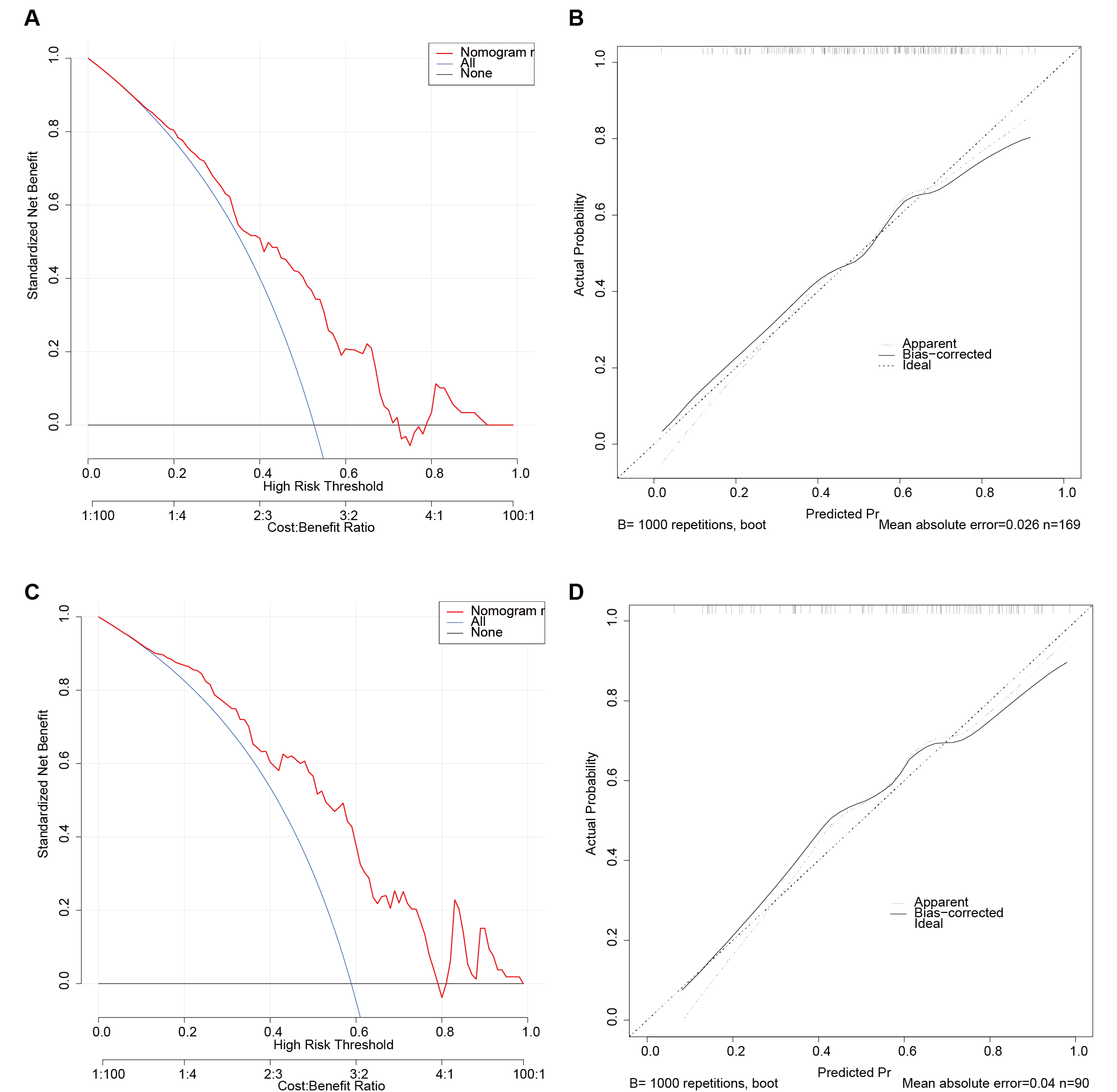

Supplement: Supplementary Figure 7 — Decision curve analysis and calibration curves for nomogram model. (A, B) DCA and Calibration curves for the nomogram model in the entire cohort. (C, D) DCA and Calibration curves for the nomogram model in the chemo-immunotherapy cohort. [file Image7.png]

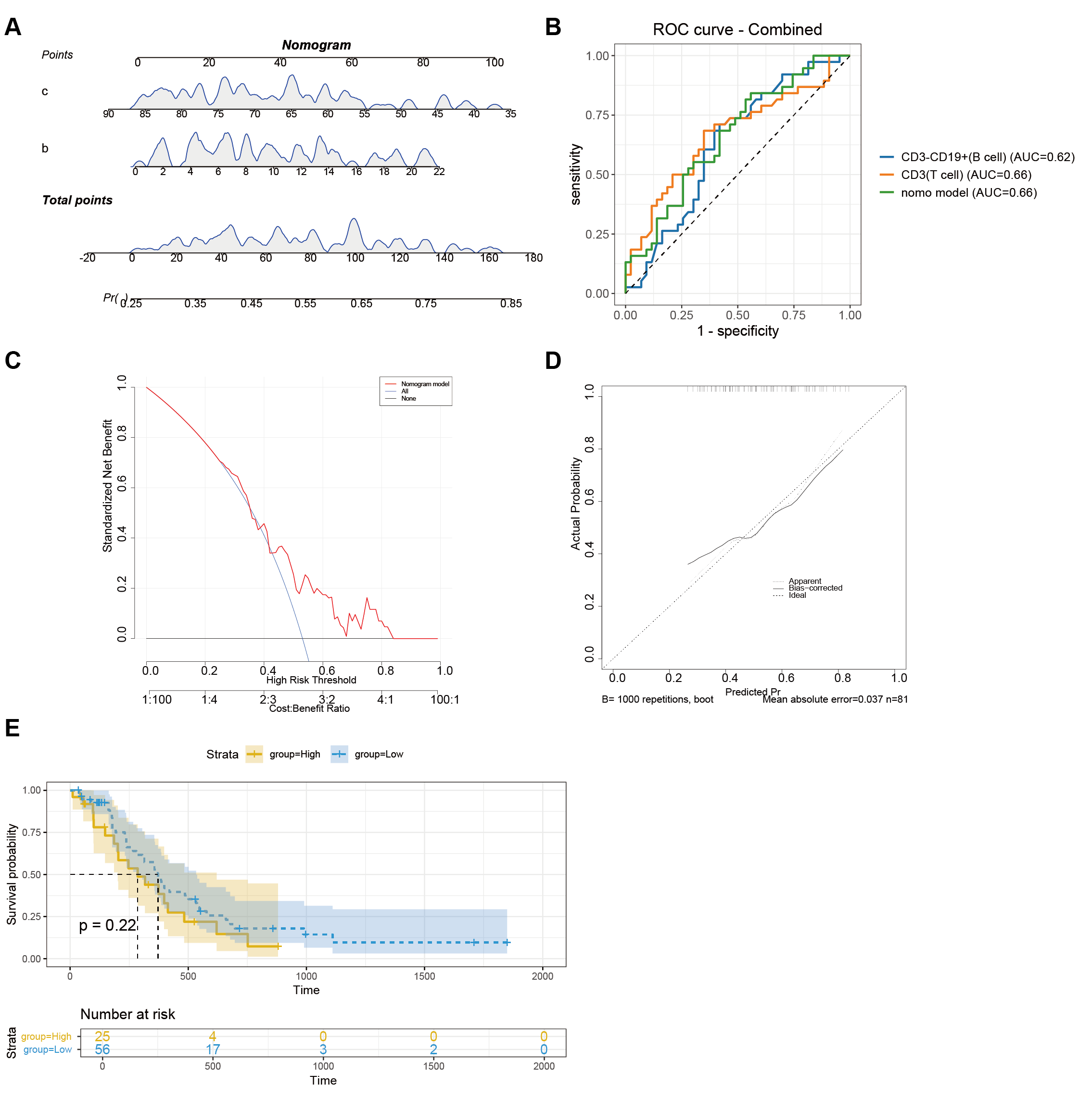

Supplement: Supplementary Figure 8 — Predictive performance of the nomogram model for chemotherapy-only patients. (A) Nomogram predicting treatment efficacy based on lymphocyte subsets in chemo-only patients. (B) The ROC curves comparing the predictive accuracy of the Nomogram model and individual risk factors. (C, D) DCA and Calibration curves of nomogram for the established nomogram. (E) Kaplan-Meier curves for PFS based on nomogram derived risk stratification. [file Image8.png]

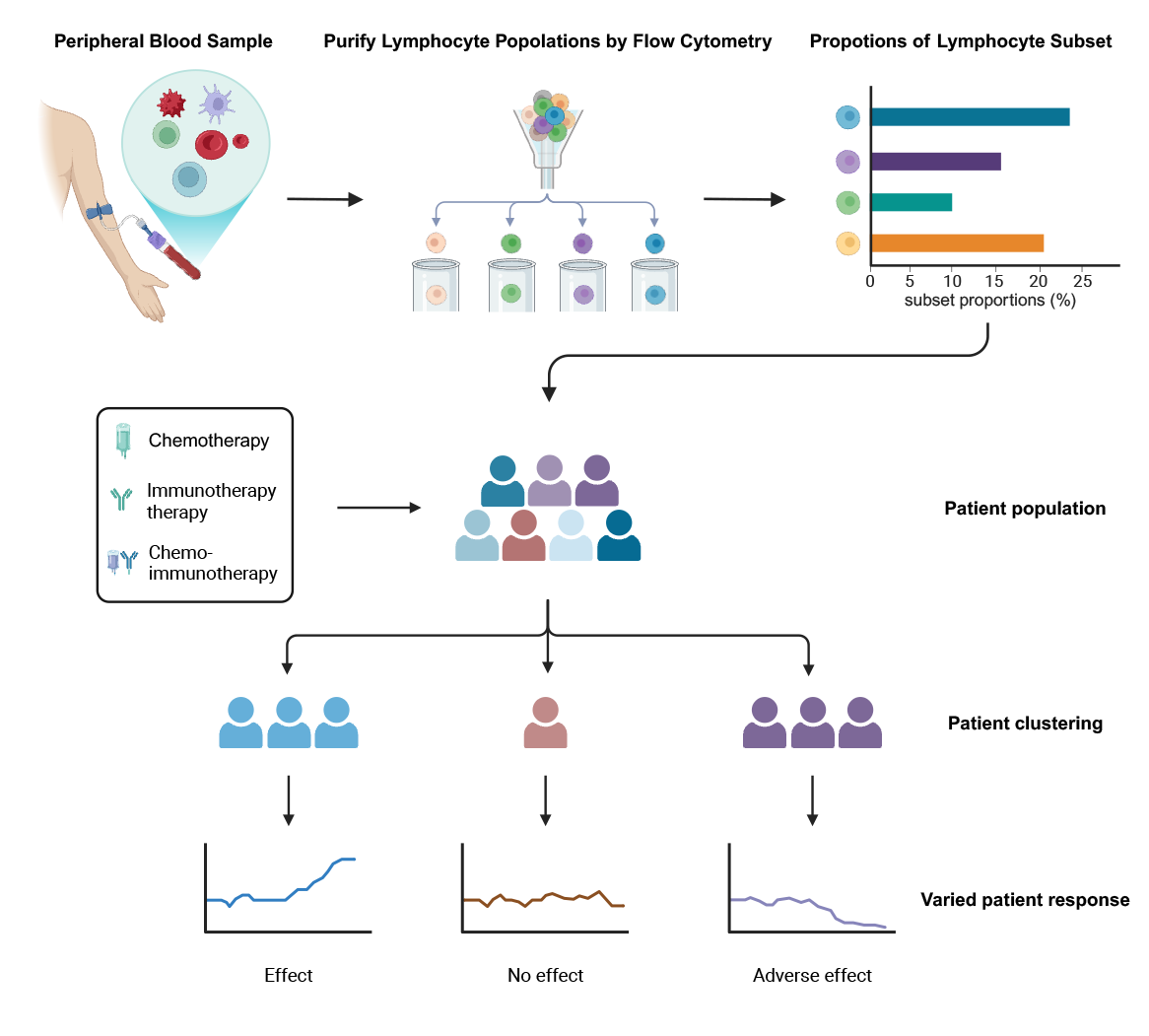

Supplement: Supplementary Figure 9 — Graphic abstract. [file Image9.tif]
