# Supplementary material for: The application of peripheral blood immune profiling in personalized treatment of locally advanced and advanced lung cancer: a nomogram approach
Source: Front Oncol. 2025 Sep 1;15:1642829. doi: 10.3389/fonc.2025.1642829 (PMC12433846; doi:10.3389/fonc.2025.1642829)
Supplement: Supplementary Table 1 — (A) Univariate Logistic regression of lymphocyte subsets for treatment efficacy in enrolled patients. (B) Multivariate Logistic regression of lymphocyte subsets for treatment efficacy in enrolled patients. [file Table1.docx]

**Supplementary Table 1A. Univariate Logistic regression of lymphocyte subsets for tretment efficacy in enrolled patients**.

| Variables | β | S.E | Z | *P* | OR (95%CI) |
| --- | --- | --- | --- | --- | --- |
|  |  |  |  |  |  |
| CD3^-^CD16^+^CD56^+^(NK cell) | -0.03 | 0.01 | -2.21 | **0.027** | 0.97 (0.94 ~ 0.99) |
| CD3^-^CD19^+^(B cell) | -0.08 | 0.03 | -2.81 | **0.005** | 0.92 (0.87 ~ 0.98) |
| CD3(T cell) | 0.06 | 0.01 | 3.94 | **<.001** | 1.06 (1.03 ~ 1.09) |
| CD3^+^CD4^+^(Th cell) | 0.00 | 0.01 | 0.18 | 0.857 | 1.00 (0.98 ~ 1.03) |
| CD3^+^CD8^+^(Tc/Ts) | 0.07 | 0.02 | 3.58 | **<.001** | 1.07 (1.03 ~ 1.11) |
| CD4^+^/CD8^+^ | -0.33 | 0.15 | -2.12 | **0.034** | 0.72 (0.53 ~ 0.97) |
| OR: Odds Ratio, CI: Confidence Interval, **Bold values indicate statistical significance (P < 0.05).** | | | | | |

**Supplementary Table 1B. Multivariate Logistic regression of lymphocyte subsets for tretment efficacy in enrolled patients.**

| Variables | β | S.E | Z | *P* | OR (95%CI) |
| --- | --- | --- | --- | --- | --- |
|  |  |  |  |  |  |
| Intercept | -12.42 | 6.17 | -2.01 | **0.044** | 0.00 (0.00 ~ 0.72) |
| CD3^-^CD16^+^CD56^+^(NK cell) | 0.11 | 0.07 | 1.69 | 0.090 | 1.12 (0.98 ~ 1.27) |
| CD3^-^CD19^+^(B cell) | 0.06 | 0.07 | 0.95 | 0.344 | 1.07 (0.93 ~ 1.22) |
| CD3(T cell) | 0.16 | 0.07 | 2.49 | **0.013** | 1.18 (1.04 ~ 1.34) |
| CD3^+^CD8^+^(Tc/Ts) | -0.02 | 0.05 | -0.40 | 0.690 | 0.98 (0.89 ~ 1.08) |
| CD4^+^/CD8^+^ | -0.51 | 0.40 | -1.26 | 0.206 | 0.60 (0.27 ~ 1.32) |
| OR: Odds Ratio, CI: Confidence Interval, **Bold values indicate statistical significance (P < 0.05).** | | | | | |
