# Supplementary material for: The application of peripheral blood immune profiling in personalized treatment of locally advanced and advanced lung cancer: a nomogram approach
Source: Front Oncol. 2025 Sep 1;15:1642829. doi: 10.3389/fonc.2025.1642829 (PMC12433846; doi:10.3389/fonc.2025.1642829)
Supplement: Supplementary Table 2 — (A) Univariate Logistic regression of lymphocyte subsets for treatment efficacy in combination patients. (B) Multivariate Logistic regression of lymphocyte subsets for treatment efficacy in combination patients. [file Table2.docx]

**Supplementary Table 2A. Univariate Logistic regression of lymphocyte subsets for tretment efficacy in combination patients.**

| Variables | β | S.E | Z | *P* | OR (95%CI) |
| --- | --- | --- | --- | --- | --- |
|  |  |  |  |  |  |
| CD3^-^CD16^+^CD56^+^(NK cell) | -0.05 | 0.02 | -2.42 | **0.015** | 0.95 (0.91 ~ 0.99) |
| CD3^-^CD19^+^(B cell) | -0.07 | 0.04 | -1.65 | 0.099 | 0.94 (0.86 ~ 1.01) |
| CD3(T cell) | 0.07 | 0.02 | 3.34 | **<.001** | 1.07 (1.03 ~ 1.12) |
| CD3^+^CD4^+^(Th cell) | -0.00 | 0.02 | -0.03 | 0.972 | 1.00 (0.97 ~ 1.03) |
| CD3^+^CD8^+^(Tc/Ts) | 0.11 | 0.03 | 3.54 | **<.001** | 1.12 (1.05 ~ 1.19) |
| CD4^+^/CD8^+^ | -0.43 | 0.21 | -2.05 | **0.040** | 0.65 (0.44 ~ 0.98) |
| OR: Odds Ratio, CI: Confidence Interval, **Bold values indicate statistical significance (P < 0.05).** | | | | | |

**Supplementary Table 2B. Multivariate Logistic regression of lymphocyte subsets for tretment efficacy in combination patients.**

| Variables | β | S.E | Z | *P* | OR (95%CI) |
| --- | --- | --- | --- | --- | --- |
|  |  |  |  |  |  |
| Intercept | -6.09 | 3.77 | -1.61 | 0.106 | 0.00 (0.00 ~ 3.68) |
| CD3^-^CD16^+^CD56^+^(NK cell) | 0.02 | 0.04 | 0.52 | 0.602 | 1.02 (0.94 ~ 1.11) |
| CD3(T cell) | 0.09 | 0.05 | 1.90 | 0.058 | 1.10 (1.00 ~ 1.21) |
| CD3^+^CD8^+^(Tc/Ts) | 0.03 | 0.08 | 0.33 | 0.740 | 1.03 (0.87 ~ 1.21) |
| CD4^+^/CD8^+^ | -0.49 | 0.63 | -0.78 | 0.438 | 0.61 (0.18 ~ 2.11) |
| OR: Odds Ratio, CI: Confidence Interval, **Bold values indicate statistical significance (P < 0.05).** | | | | | |
